# Identification and utilization of two important transporters: SgvT1 and SgvT2, for griseoviridin and viridogrisein biosynthesis in *Streptomyces griseoviridis*

**DOI:** 10.1186/s12934-017-0792-8

**Published:** 2017-10-25

**Authors:** Yunchang Xie, Junying Ma, Xiangjing Qin, Qinglian Li, Jianhua Ju

**Affiliations:** 1 CAS Key Laboratory of Tropical Marine Bioresources and Ecology, Guangdong Key Laboratory of Marine Materia Medica, Research Network for Applied Microbiology Center for Marine Microbiology, South China Sea Institute of Oceanology, Chinese Academy of Sciences, Guangzhou, 510301 China; 2 College of Earth Sciences, University of Chinese Academy of Sciences, Beijing, 10049 China

**Keywords:** Metabolite transporter, SgvT1, SgvT2, Griseoviridin, Viridogrisein, Streptogramin, Antibiotic biosynthesis, *Streptomyces griseoviridis*

## Abstract

**Background:**

Griseoviridin (GV) and viridogrisein (VG, also referred as etamycin), both biosynthesized by a distinct 105 kb biosynthetic gene cluster (BGC) in *Streptomyces griseoviridis* NRRL 2427, are a pair of synergistic streptogramin antibiotics and very important in treating infections of many multi-drug resistant microorganisms. Three transporter genes, *sgvT1*–*T3* have been discovered within the 105 kb GV/VG BGC, but the function of these efflux transporters have not been identified.

**Results:**

In the present study, we have identified the different roles of these three transporters, SgvT1, SgvT2 and SgvT3. SgvT1 is a major facilitator superfamily (MFS) transporter whereas SgvT2 appears to serve as the sole ATP-binding cassette (ABC) transporter within the GV/VG BGC. Both proteins are necessary for efficient GV/VG biosynthesis although SgvT1 plays an especially critical role by averting undesired intracellular GV/VG accumulation during biosynthesis. SgvT3 is an alternative MFS-based transporter that appears to serve as a compensatory transporter in GV/VG biosynthesis. We also have identified the γ-butyrolactone (GBL) signaling pathway as a central regulator of *sgvT1*–*T3* expression. Above all, overexpression of *sgvT1* and *sgvT2* enhances transmembrane transport leading to steady production of GV/VG in titers ≈ 3-fold greater than seen for the wild-type producer and without any notable disturbances to GV/VG biosynthetic gene expression or antibiotic control.

**Conclusions:**

Our results shows that SgvT1–T2 are essential and useful in GV/VG biosynthesis and our effort highlight a new and effective strategy by which to better exploit streptogramin-based natural products of which GV and VG are prime examples with clinical potential.

**Electronic supplementary material:**

The online version of this article (doi:10.1186/s12934-017-0792-8) contains supplementary material, which is available to authorized users.

## Background

**Fig. 1:**
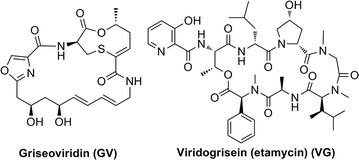
Chemical structures of griseoviridin (GV) and viridogrisein (VG)

The actinomycete *Streptomyces griseoviridis* NRRL 2427 generates two types of unrelated streptogramins; these include griseoviridin (GV) belonging to the A-type cyclic polyunsaturated macrolactone subclass, and viridogrisein (VG), a B-type cyclic depsipeptide counterpart to GV also referred to as etamycin (Fig. 1) [1, 2]. GV and VG bind to the A and P sites, respectively, of the 50S bacterial ribosomal subunit thereby averting peptide bond formation during the elongation phase of protein translation. Moreover, GV and VG have been noted to work synergistically leading to antibacterial activities superior to those of either compound alone; this effect suggests that the combination of GV/VG has great potential with respect to treating multidrug resistant microbial pathogens [3–5].

Stemming from the diversity of structures and bioactivities of their secondary metabolites, the streptogramin-producing actinomycetes commonly employ several transmembrane transporters as drug efflux pumps to avoid intracellular metabolite accumulation. These transporters typically belong to major facilitator superfamily (MFS) and ATP-binding cassette (ABC) families and constitute an essential self-resistance mechanism to efficiently secrete antibiotics as they are constructed thereby protecting the producing microbe from the effects of its own secondary metabolism [5–10]. This is a central idea in microbial homeostasis [11–14]. MFS transporters commonly possess 12 or 14 transmembrane segments (TMS) and employ a transmembrane electrochemical gradient to secrete secondary metabolites [11–15]. Conversely, the ABC transporters share a conserved domain organization of separate transmembrane domain (TMD) subunits and nucleotide-binding domain (NBD, also called ATP-binding cassette) subunits enabling ATP hydrolysis and application of the resulting energy to effect metabolite removal from the cell [11–14, 16]. Notably, the majority of prokaryotic ABC transporters consist of stand-alone TMD or NBD polypeptides which must dimerize in some fashion to generate fully functional proteins [12, 17].

**Fig. 2:**
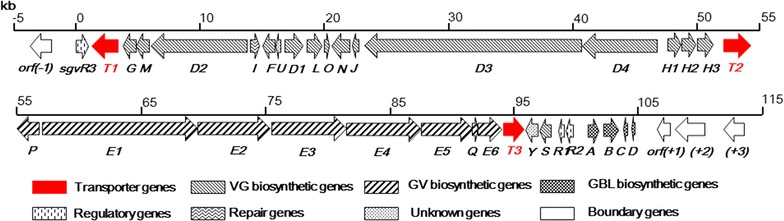
Gene organization of the GV VG gene cluster in *S. griseoviridis* NRRL 2427. The direction of transcription and the proposed functions of individual ORFs are indicated

Not surprisingly, overexpression of actinomycete efflux transporters significantly enhances antibiotic release rates. This translates to enhanced antibiotic production by reducing feedback inhibition in vivo [13, 18]. This strategy, termed “transporter engineering” is widely applied in large-scale industrial antibiotic production processes; some examples include production of pradimicin, avermectin, doxorubicin and neomycin in *Streptomyces* [19–22]. Enabled by the recently identified 105-kb GV/VG biosynthetic gene cluster (Fig. 2) [1, 2], we report herein: (i) the identification of three transporter encoding genes housed within the GV/VG biosynthetic gene cluster: *sgvT1*–*T3* (SgvT1 and SgvT3 are MFS transporters and SgvT2 is an ABC transporter) that are regulated by γ-butyrolactone (GBL)-type signaling, (ii) SgvT1 and SgvT2 are both necessary for efficient GV/VG biosynthesis with SgvT1 playing an indispensable role in maintaining stable expression throughout sustainable GV/VG biosynthesis, and (iii) a roughly threefold increase in GV/VG titers resulting from *sgvT1*–*T2* overexpression. These studies provide new insight into host self-resistance mechanisms in streptogramin biosynthesis and highlight a practical approach to improving streptogramin production.

## Results

### Discovery of SgvT1–T3 as a two class-based transporter system

*Streptomyces griseoviridis* NRRL 2427 is a well-known producer of GV and VG; the highest yield of GV is 33.04 ± 0.70 μg/mL and that of VG is 31.56 ± 0.51 μg/mL both over the course of 108 h fermentation. Subsequent early analyses confirmed that ≈ 75% of GV/VG produced in such systems is found in the fermentation supernatant (Additional file 1: Table S6) suggesting that *S*. *griseoviridis* NRRL 2427 houses an effective efflux system in accordance with the annotated set of transporters genes *sgvT1*–*T3* (Fig. 2).

The gene *sgvT1* encodes a 531 aa MFS type transporter and is located proximal to the upstream boundary of the *sgv* biosynthetic gene cluster. Conversely, *sgvT3* which codes for another MFS type transporter composed of 464 aa, is positioned downstream of *sgvT1* and proximal to the 10 kb regulatory region within the *sgv* gene cluster (Fig. 2) [1]. The assignment of SgvT1 and SgvT3 as MFS type proteins is based on the alignment of conserved sequences and domains with previously characterized acinomycete MFS transporters, such as CmcT (*Amycolatopsis lactamdurans* and *S*. *clavuligerus*), EncT (*S. maritimus*) and MctT (*S. lavendulae*) [11, 23–25]. Both SgvT1 and SgvT3 possess TMS for functional exploitation of transmembrane electrochemical gradients [14]. SgvT3 contains 14 highly conserved TMS regions whereas SgvT1 contains 15 TMS fragments with two different regions and one more TMS region in the C terminal, even though the other 13 TMS are quite conserved (Additional file 1: Figure S1). By contrast, SgvT1 is slightly larger than many of its counterpart transporters by an average of ≈ 50–70 aa; this increased size may explain the additional fold seen with SgvT1 but not in related counterparts.

The *sgvT2* gene represents the point of differentiation between upstream VG biosynthesis and downstream GV biosynthesis components and encodes the only 551 aa ABC transporter (Fig. 2) [1]. Notably, the product SgvT2 is composed of two NBD domains but is devoid of any TMD domain [1]. On the basis of conserved domain assays using actinomycete-derived and rigorously characterized NBD-containing ABC transporters (devoid of TMDs), such as AvtA (*S*. *avermitilis*), DrrA (*S*. *peucetius*), KasK (*S*. *kasugaensis*), MtrA (*S*. *argillaceus*), OleC (*S*. *antibioticus*) and PdmR1 (*Actinomadura hibisca*) [19, 20, 26–29], the two SgvT2 NBDs can be defined as SgvT2-Fr (1–240/240 aa) and SgvT2-Re (365–520/156 aa), respectively. The SgvT2-Fr contains all three catalytic ATP-hydrolyzing sequences termed Walker A, Walker B and “signature” motifs. SgvT2-Fr also contains one additional D-loop region. Interestingly, SgvT2-Re also contains the Walker B and signature motifs but is devoid of the critical Walker A motif (Additional file 1: Figure S2). Quite significantly, we could not identify any TMD domain-type transporters within the GV/VG biosynthetic gene cluster. Consequently, it is quite likely that the potential dimerized partner(s) resides beyond the gene cluster.

### Identification of SgvT1/T2 as two necessary transporters in regular GV/VG biosynthesis

**Fig. 3:**
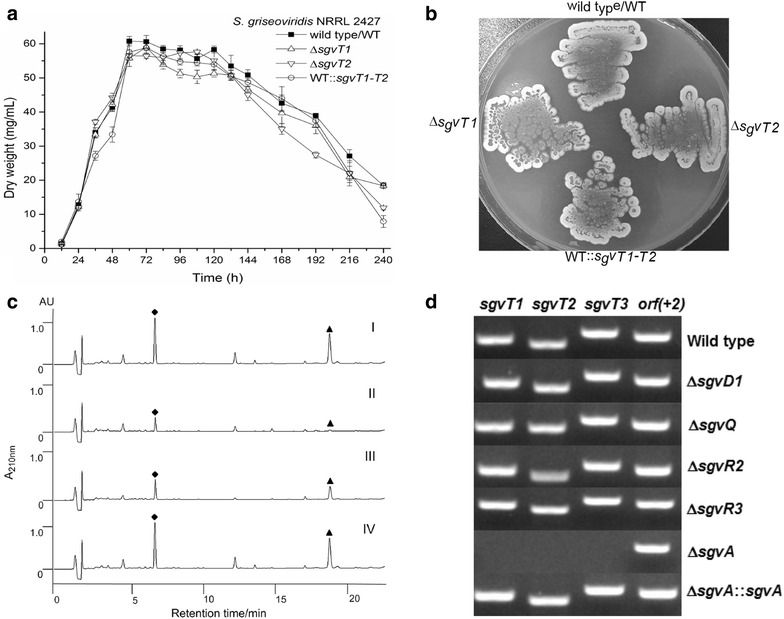
Comparisons of different strain growth patterns, plate-cultured morphologies, fermentation and gene expression profiles. **a** Growth curve of *S. griseoviridis* NRRL 2427 wild-type (WT) strain, Δ*sgvT1/T2* mutants and WT strain complemented with *sgvT1*–*T2*; **b** The cultured morphology of wild-type strains, Δ*sgvT1/T2* mutants and WT strain complemented with *sgvT1*–*T2* in M-ISP4 medium plate; **c** HPLC analysis of GV (black diamond) and VG (black up-pointing triangle) in fermentation extract. (I) wild-type strain, (II) Δ*sgvT1* mutant, (III) Δ*sgvT2* mutant, (IV) Δ*sgvT3* mutant; **d** RT-PCR analysis of *sgvT1*–*T3* and control boundary gene *orf (*+*2)* in wild-type strain and Δ*sgvD1*, Δ*sgvQ*, Δ*sgvR2*, Δ*sgvR3*, Δ*sgvA* mutants and Δ*sgvA*::*sgvA* strain

**Fig. 4:**
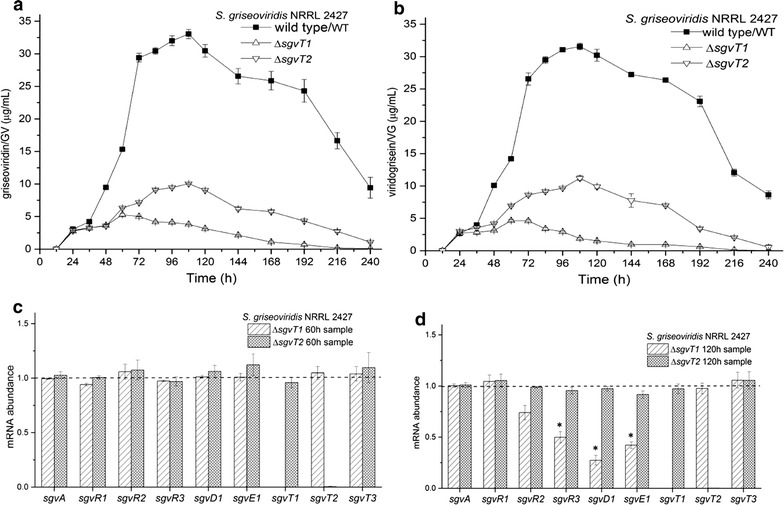
Comparison of GV/VG production and gene expression profiles in different strains. The GV (**a**) and VG (**b**) production curves in *S. griseoviridis* NRRL 2427 wild-type (WT) strain, Δ*sgvT1/T2* mutants; The qPCR analysis of GV/VG biosynthesis-related gene expression during fermentation of Δ*sgvT1/T2* mutants at 60 h (**c**) and 120 h **(d**) (*p < 0.05), the dash line indicate the mRNA abundance level of WT in the same conditions

Using established λ-RED-mediated PCR-targeting mutagenesis methods we constructed gene insertion mutants Δ*sgvT1*–*T3* for subsequent function analyses. Following validation of the desired mutations, all three mutant strains were cultured in liquid or solid medium and no obvious changes could be noted relative to the wild-type strain based on growth curves and morphological analyses (Fig. 3a, b). The constructed mutant strains displayed significantly different efficiencies in GV/VG production (Fig. 3c). GV/VG production by the Δ*sgvT1* mutant strain was significantly impaired; metabolite generation (optimal) by this strain was only 14–16% of that realized with WT producer [yields: (5.28 ± 0.19 μg/mL for GV and 4.65 ± 0.13 μg/mL for VG after 60 h fermentation)]. The Δ*sgvT2* strain also suffered sharply reduced GV/VG production efficiencies with yields only on par with 30–35% of that observed with WT producer; metabolite titers for the Δ*sgvT2* were 10.05 ± 0.09 μg/mL GV and 11.21 ± 0.35 μg/mL VG after fermentation for 108 h (see: Fig. 4a, b and Additional file 1: Table S6). Importantly, the highest intracellular GV/VG concentrations in the Δ*sgvT1* and Δ*sgvT2* mutant strains were found to be ≈ 1.5 μg/mL. Intracellular GV/VG concentrations for the WT producer proved substantially higher at 8.12 ± 0.62 μg/mL GV and 7.12 ± 0.52 μg/mL VG using comparable fermentation conditions (Additional file 1: Table S6). The dramatically different intracellular concentrations for GV/VG between mutant and the unmodified WT strains suggest impaired biosynthesis correlating to *sgvT1* and *sgvT2* inactivation indicating that the encoded transporters are integral to efficient GV/VG biosynthesis. Then, we performed the *trans* complementation of Δ*sgvT1* and Δ*sgvT2* mutant strains and compared the fermentation with WT strains. As expected, we found that both two trans complemented stains’ GV/VG biosynthesis could be readily restored to near WT levels (Additional file 1: Figure S11). These complementation assay also verified the indispensable roles of SgvT1 and SgvT2 in GV/VG biosynthesis, in turn.

By contrast, little to no impact on GV/VG titers were apparent following metabolite analysis of Δ*sgvT3* strain fermentations (Fig. 3c). This finding indicates that the SgvT3 maybe a compensatory transporter responsible for transport of intermediates in GV/VG biosynthesis. Moreover, it is interesting to note that, whereas inactivation of SgvT1/T2 had a profound influence on GV/VG production, inactivation of SgvT3 had no influence upon metabolite titers. Thus, this finding also suggests the alternative role of SgvT3 in the transporter of GV/VG, which can likely be carried out by the intact SgvT1/T2 system.

### Regulation of *sgvT1*–*T3* expression by the GBL signaling pathway

**Fig. 5:**
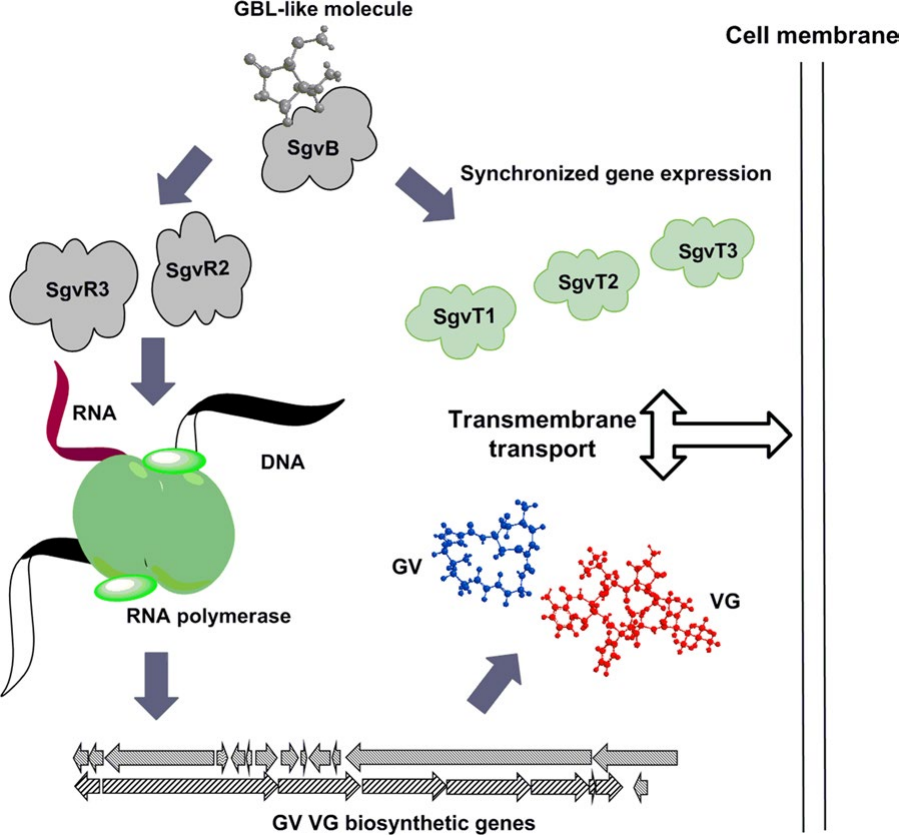
Proposed transfer mechanism via SgvT1–T3 in GV/VG biosynthesis in *S*. *griseoviridis* NRRL 2427. GBL signal pathway not only activates the GV/VG biosynthesis but also prompts the synchronized *sgvT1*–*T3* expression to avoid disorder GV/VG accumulation

Global on/off regulation via γ-butyrolactone (GBL)-type “quorum sensing” signal molecules of pathway-specific activation of GV/VG biosynthesis has been previously shown [1]. Inactivation of the essential GBL signal molecule biosynthetic gene *sgvA* will effectively shut down expression of GV/VG biosynthetic pathway-specific activators coding genes *sgvR2* and sgv*R3*. Consequently, inactivation of either *sgvR2* or *sgvR3* leads to abolished expression of GV and VG biosynthetic genes such as *sgvQ* and *sgvD1*, respectively. This cascade of gene inactivations ultimately precludes GV/VG biosynthesis [1]. Using a Δ*sgvA* mutant devoid of GBL signaling capacity we found that *sgvT1*–*T3* all failed to be expressed, which could be restored in strain Δ*sgvA*::*sgvA* (*sgvA* insertion mutant Δ*sgvA* complemented with *sgvA*) (Fig. 3d). However, mutant strains Δ*sgvR2* and Δ*sgvR3* in which regulatory genes had been inactivated, as well as strains in which key GV/VG backbone biosynthesis genes were inactivated (Δ*sgvQ* and Δ*sgvD1*) were all found to express *sgvT1*–*T3* at rates similar to that seen with the WT producer (Fig. 3d). On the basis of these data it is clear that *sgvT1*–*T3* expression is tightly regulated by global GBL signaling and is not significantly or directly influenced by GV/VG biosynthetic machinery (Fig. 5).

### Identification of the pivotal role of SgvT1 in sustainable GV/VG biosynthesis

Analysis of GV/VG production efficiencies (Fig. 4) reveals that Δ*sgvT1* and Δ*sgvT2* mutant strains both produce GV and VG with comparable efficiencies up to approx. 48 h. However, differences in biosynthetic efficiencies between the two strains become noticeable starting at about 48 h and continuing to 120 h where the difference is most pronounced (Fig. 4a, b). In efforts to correlate GV/VG biosynthesis to specific changes in gene expression in both Δ*sgvT1* and Δ*sgvT2* mutants we employed qPCR to monitor changes in mRNA levels for specific GV/VG-related genes. These included: *sgvA* (putative GBL signaling gene), *sgvR1* (repressor gene), *sgvR2, R3* (activator genes), GV/VG backbones biosynthetic genes *sgvE1*/*sgvD1* (GV/VG backbone biosynthetic genes), and *sgvT1*–*T3* [1]. We found that transcripts for Δ*sgvT2* correlated very closely with those found for WT strain at during the early (up to 60 h) phases of GV/VG biosynthesis. This was the case also for the intermediate (120 h) stage of biosynthesis presumably because intact SgvT1 and SgvT3 could ensure GV/VG transmembrane transport needed to prevent intracellular accumulation of GV and/or VG (Fig. 4c, d) and subsequent feedback inhibition. Similarly, transcript analyses for the Δ*sgvT1* mutant revealed normal levels of gene expression in the early stage of GV/VG biosynthesis on par with those observed for Δ*sgvT2* and WT strains (Fig. 4c).

In considering GV/VG biosynthesis over the course of 120 h we noted that all GV/VG biosynthetic genes, except for *sgvA*, *sgvR1* and *sgvT3*, were down-regulated in the Δ*sgvT1* mutant (Fig. 4d). These changes indicated that only intact SgvT2 and SgvT3 could not potentially sustain GV/VG biosynthesis throughout all fermentation stages. In concert with the realization (Fig. 4a, b) that, over this time frame, GV/VG titers are most dramatically different between the Δ*sgvT1* and Δ*sgvT2* mutants, it becomes clear that SgvT1 plays an apparently critical role in modulating GV/VG biosynthesis and that its effects correlate to changes in *sgvR2*, *sgvR3*, *sgvD1*, and *sgvE1*. That no comparable decreases in transcripts were noted for the sgvT2 mutant (Fig. 4c, d) suggests SgvT2 is important as one element of a SgvT2/T3 efflux system but that its role in this manifold is secondary to that of the SgvT1/T2 system. These data firmly suggest that effective GV/VG biosynthesis in *S. griseoviridis* NRRL 2427 calls for an intact SgvT1/T2 efflux system and that the effectiveness of this transport mechanism far exceeds that of a putative SgvT2/T3 system.

### Generation of a GV/VG high yielding recombinant strain by overexpression of *sgvT1/T2* in *S. griseoviridis* NRRL 2427

**Fig. 6:**
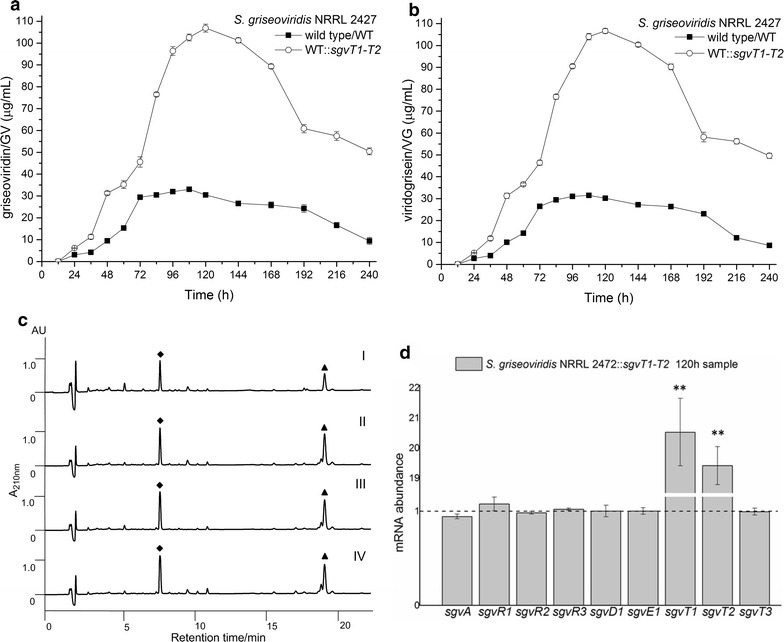
Analysis of GV/VG high yielding recombinant strain WT::*sgvT1*–*T2*. The GV (**a**) and VG (**b**) production curves; **c** the HPLC analysis of GV (black diamond) and VG (black up-pointing triangle) in fermentation extract. (I) wild-type (WT) strain, (II–IV) twofold diluted sample of WT::*sgvT1*-*T2*; **d** the qPCR analysis of GV/VG biosynthetic gene expression profiles during fermentation of WT::*sgvT1*–*T2* (**p < 0.01), the dash line indicate the mRNA abundance level of WT in the same conditions

Given the importance of the SgvT1/T2 efflux system we sought to generate a high yielding GV/VG-producing strain via transport engineering. Notably, the *trans* complementation in Δ*sgvT1* and Δ*sgvT2* mutant strains could readily restore the GV/VG to near WT levels (Additional file 1: Figure S11). Predicated on this finding, and the clear importance of SgvT1/T2 efflux we envisioned that sgvT1/T2 over-expression in the WT strain might readily afford a high yielding GV/VG producer. Accordingly *sgvT1/T2* were incorporated into the WT producer to afford *S. griseoviridis* NRRL 2427::*sgvT1*–*T2* (WT::*sgvT1*–*T2*). Placement under the control of the *ermE* promoter afforded approximately a 20-fold over-expression of *sgvT1* and a 19-fold over-expression of *sgvT2*. As expected, the high-yielding WT::*sgvT1*–*T2* strain was found to generate GV/VG in substantially greater yield than does the native *S. griseoviridis.* Commensurate with expectation, GV and VG yields relative to the WT producer increased by ≈ 3-fold [(106.85 ± 1.81 μg/mL GV/106.61 ± 1.12 μg/mL VG) see Fig. 6a–c, Additional file 1: Table S6]. Especially noteworthy is that GV/VG yields steadily increased while the expression of previously examined biosynthetic genes (Fig. 4c, d) remained constant; the latter is indicative of stable intracellular homeostasis (Fig. 6d). Additionally, we found that the higher yields of GV and VG obtained using WT::*sgvT1*–*T2* were achieved even in the absence of antibiotic generally used to maintain selective pressure (Additional file 1: Figure S10).

## Discussion

Transporter-encoding genes within secondary metabolite biosynthesis gene clusters are central to cell viability and homeostasis; both rely heavily on a cell’s ability to pump antibiotics and other toxic metabolites out of the cell [11–14]. Advances in genomics and gene inactivation assays enabled us to identify three transporter encoding genes: *sgvT1*–*T3* distributed within the GV/VG biosynthetic gene cluster. SgvT3 is a conserved MFS transporter with 14 TMS. Interestingly, inactivation of *sgvT3* failed to significantly impact GV/VG titers suggesting that SgvT3 may serve as a form of alternative transporter. Precedence for this logic has been reported for landomycin C and cephalosporin; inactivations of *lndW* (in the landomycin cluster) and *cefT* (in the cephalosporin cluster) also failed to impact natural product titers [30, 31]. The failure of inactivations of putative transporters to significantly alter metabolite titers suggests that losses in their activity can be compensated for using substitute or alternative transport mechanisms. In the case of GV/VG biosynthesis, we found that loss of SgvT3 activity is likely compensated for by its counterparts SgvT1 and SgvT2.

SgvT1 and SgvT2 are both necessary transporters involved in GV/VG biosynthesis; inactivation of either protein significantly decreased GV/VG fermentation titers. SgvT1 is a rare 15 TMS-containing MFS transporter. Alternatively, SgvT2 is composed of two NBD domains but is devoid of any TMS. The absence of TMS elements in SgvT2 suggests a reliance upon other TBD domain-containing transporters in order for SgvT2 to be involved in any kind of effective GV/VG efflux system. That two distinct chemical species (GV and VG) are generated by the *S. griseoviridis* producer also supports the notion that effective efflux systems may require two dramatically different types of transporter proteins to work synergistically to maintain homeostasis within the producer.

Griseoviridin and VG are both excellent examples of streptogramins and previous work has placed a great deal of importance on understanding mechanisms of streptogramin cellular export during biosynthesis [4–10]. We focus herein much more intensely on SgvT1 and SgvT2. Careful analyses of both metabolite production profiles and mRNA levels throughout long periods up to 240 h of fermentation unveiled a new appreciation for the importance of SgvT1 in GV/VG biosynthesis. In particular, the careful tracking of both GV/VG titers and transcript levels for selected key genes using Δ*sgvT1* and Δ*sgvT2* mutant strains shed tremendous insight into the roles of these transporter genes. These analyses make clear that SgvT1 is pivotal to metabolite export and that its inactivation is only minimally compensated for by either SgvT2/T3 or perhaps other transporters within the producer genome. This is in stark contrast to the case of *sgvT3* inactivation, which, based on our results, is readily compensated for by an intact SgvT1/T2 transport couple.

Inactivations of *sgvT1*–*T3* do not appear to directly affect the expression of GV/VG biosynthetic or activating genes. Only the Δ*sgvT1* mutant appeared to have any of these gene’s expression indirectly inhibited, presumably in response to intracellular environmental adaptations. Moreover, we found that *sgvT1*–*T3* are regulated by the GBL signaling pathway, which appears to synchronize with GV/VG biosynthesis without directly influencing GV/VG titers. On the basis of these findings we envision that, when the *S. griseoviridis* (WT or mutants) producer receives the GBL signal to activate GV/VG biosynthesis, *sgvT1*–*T3* expression ensues to coordinate prompt GV/VG secretion thereby averting intracellular GV/VG accumulation. In this way, we posit that GBL signaling plays a key role in GV/VG biosynthesis by maintaining cellular homeostasis and coordinating GV/VG biosynthesis and transmembrane export (Fig. 5).

An overarching goal of transport engineering is to increase natural product titers via overexpression of transport genes. Accordingly, and motivated by our findings with *sgvT1*–*T3*, we overexpressed *sgvT1* and *sgvT2* to enhance transmembrane secretion of GV/VG thereby improving natural product titers. Exploitation of *ermE* promoter technology enabled us to overexpress both *sgvT1*/*T2* by ~ 20-fold which translated to a threefold increase in GV and VG titers. Notably, this approach does not disturb biosynthetic gene expression profiles and affords a producer no longer dependent on environmental antibiotic exposure to generate the antimicrobials of interest. Over-expression of *sgvT1*/*T2* in the mutant GV/VG producer not only validates the importance of these transporters but also provide a new approach for future industrialized exploitation and use of the streptogramins GV and VG.

## Conclusions

In this study we have identified the roles of SgvT1–T3 in GV/VG biosynthesis: SgvT1 and SgvT2 are both necessary for efficient GV/VG biosynthesis with SgvT1 playing an indispensable role in maintaining stable biosyntheic gene expression throughout sustainable GV/VG biosynthesis; but SgvT3 is an alternative MFS-based transporter that appears to serve as a compensatory protein in GV/VG biosynthesis. All these three transporters’ coding genes are regulated by γ-butyrolactone (GBL)-type signaling, which appears to synchronize with GV/VG biosynthesis and avert excessive intracellular GV/VG accumulation. Enabled by the discovery and application of two critical transporters SgvT1/T2, we constructed a new steady GV/VG high-producing strain in titers about threefold greater than seen for the wild-type producer and without any notable disturbances to GV/VG biosynthetic gene expression or environmental antibiotic control. Consequently, our effort highlighted a new strategy to better exploit streptogramin-based natural products and demonstrate that enhancing self-resistance mechanisms in antimicrobial producing organisms is a valuable approach to safely and efficiently improving metabolite production processes.

## Methods

### Bacterial strains, plasmids, media and general experimental procedures

The general methods and materials used, as well as, all bacterial strains, plasmids, and culture conditions have all been previously described [1, 2]. Fermentations and GV/VG production and HPLC analyses of *S*. *griseoviridis* NRRL 2427 and related mutant strains were executed as previous described [1, 2].

### Gene inactivation and complementation of *sgvT1*–*T3* in *S*. *griseoviridis* NRRL 2427 wild-type (WT) and mutant strains

A λ-RED mediated PCR-targeting mutagenesis method was chosen for the inactivation of *sgvT1*–*T3* in *S*. *griseoviridis* NRRL 2427 to construct the corresponding three mutants Δ*sgvT1*–*T3* (Additional file 1: Figures S3–S5) [1, 2, 32]. The primers employed in these studies are listed in Additional file 1: Tables S1 and S2.

The *trans* complementants of Δ*sgvT1/T2* mutants were constructed using modified vector pSET152AKE, the manipulations of pSET152AKE have been previously described [1, 2]. The complementant of *S*. *griseoviridis* NRRL 2427 WT strain (WT::*sgvT1*–*T2*) was constructed by two steps. The first step entailed introduction of *sgvT1* by conjugation with pPWW50Apr-*sgvT1* and screening via apramycin resistance as previous described [33]. The *sgvT2* gene was then introduced by conjugation to pSET152AKE-*sgvT2* and screening for desired fusions was carried out by kanamycin resistance screening as previous described [1, 2]. The primers used are shown in Additional file 1: Table S3.

### Quantitative analysis of bacterial growth and GV/VG production using *S*. *griseoviridis* NRRL 2427 WT and mutant strains

Four strains, the *S*. *griseoviridis* NRRL 2427 WT strains, two mutants Δ*sgvT1/T2* and the WT::*sgvT1*–*T2*, were fermented and subsequent metabolite analyses carried out as previously described [1, 2]. Ten flasks per strain were withdrawn every 12 h to measure cell dry weights needed to construct growth curves and to determine GV/VG yields. The cell cultures (200 μL/per flask) were collected on filter papers and dried at 55 °C before weight measurements were taken.

To measure GV/VG production averages included in the confidence interval, two standard curves were generated using analytically pure sample and HPLC analysis (Additional file 1: Figure S10). To establish the GV/VG fermentation curve, ten flasks/per strain of cell cultures (250 μL/per flask) were extracted with butanone and concentrated in vacuo affording oil residues. All residues were then dissolved in MeOH and subjected to HPLC analysis. To analyze extra/intracellular GV/VG production efficiencies, each 50 mL batch of fermentation broth was centrifuged (4000*g*, 10 min) to separate precipitates and cells from the supernatant [1]. Broths were then extracted with 100 mL EtOAc and the resulting organic extracts concentrated under reduced pressure to afford residues that were then dissolved into 2 mL MeOH. Samples were then centrifuged at 12,000*g* for 15 min; both the supernatants and pellet contents were then subjected to HPLC analysis.

### RT-PCR and qPCR analysis of *S. griseoviridis* NRRL 2427 WT and mutant strains

Mycelia harvested at specific time points were powderized using in N_2_ (l) and total RNA was extracted from the frozen pellet using the SV total RNA purification Kit (Promega). RNA samples were subjected to Dnase I (Promega) digestion according to the manufacturer instructions. First-strand cDNA synthesis was accomplished using Invitrogen’s SuperScript™ Kit and second step PCR was carried out under previously indicated conditions [1]. Control RT-PCR was similarly performed in the absence of reverse transcription to check for DNA contamination after Dnase I digestion required during RNA preparation. The qPCR was performed using MaximaTM SYBR Green qPCR Mix (MBI) and Applied Biosystem’s 7500 Fast Real-time PCR system. 16S rDNA was used as the internal control. All of the primers used are shown in Additional file 1: Tables S4 and S5. All qPCR assays were repeated in triplicate. Statistical analysis was carried out using SPSS version 13. One-vay ANOVA at a 95% confidence level (*p* < 0.05 and *p* < 0.01) was used to evaluate the significance of the difference between each sample.

## Additional file

**Additional file 1: Figure S1.** The multiple alignment of SgvT1/T3 with other transporters. **Figure S2.** The multiple alignment of SgvT2 with other transporters. **Figures S3–S5.** The inactivation of *sgvT1-T3.* **Figure S6–S8.** HPLC analyses of the fermentation extract of Wild-type & Δ*sgvT1-T3*. **Figure S9.** HPLC analyses of the fermentation extract of WT::*sgvT1–T2.* **Figure S10.** The HPLC standard curve of GV/ VG. **Figure S11.** HPLC analyses of fermentation extract of complemented mutants. **Table S1.** Primer pairs used for PCR-targeting of *sgvT1–T3.* **Table S2.** Primers used for PCR confirmation of double-crossover mutants. **Table S3.** Primer pairs used for complementation of *sgvT1–T3*. **Table S4.** Primer pairs used for RT-PCR. **Table S5.** Primer pairs used for qPCR. **Table S6.** Quantitative analysis of GV/VG production.

## Abbreviations

GV: griseoviridin
VG: viridogrisein
BGC: biosynthetic gene cluster
MFS: major facilitator superfamily
ABC: ATP-binding cassette
GBL: γ-butyrolactone
TMS: transmembrane segments
TMD: transmembrane domain
NBD: nucleotide-binding domain
WT: wild-type
RT-PCR: reverse transcription PCR
qRCP: quantitative real-time PCR
