# Supplementary material for: Identification and utilization of two important transporters: SgvT1 and SgvT2, for griseoviridin and viridogrisein biosynthesis in Streptomyces griseoviridis
Source: Microb Cell Fact. 2017 Oct 25;16:177. doi: 10.1186/s12934-017-0792-8 (PMC5655939; doi:10.1186/s12934-017-0792-8)
Supplement: Supplementary file 1 — Additional file 1: Figure S1. The multiple alignment of SgvT1/T3 with other transporters. Figure S2. The multiple alignment of SgvT2 with other transporters. Figures S3–S5. The inactivation of sgvT1-T3. Figure S6–S8. HPLC analyses of the fermentation extract of Wild-type & ΔsgvT1-T3. Figure S9. HPLC analyses of the fermentation extract of WT::sgvT1–T2. Figure S10. The HPLC standard curve of GV/ VG. Figure S11. HPLC analyses of fermentation extract of complemented mutants. Table S1. Primer pairs used for PCR-targeting of sgvT1–T3. Table S2. Primers used for PCR confirmation of double-crossover mutants. Table S3. Primer pairs used for complementation of sgvT1–T3. Table S4. Primer pairs used for RT-PCR. Table S5. Primer pairs used for qPCR. Table S6. Quantitative analysis of GV/VG production. [file 12934_2017_792_MOESM1_ESM.doc]

**Identification and Utilization of Two Important Transporters: SgvT1 and SgvT2, for Griseoviridin and Viridogrisein Biosynthesis in *Streptomyces griseoviridis***

**Additional Information**

Content

| 1 | **Fig. S1** The multiple alignment of SgvT1/T3 with other transporters. | S2–3 |
| --- | --- | --- |
| 2 | **Fig. S2** The multiple alignment of SgvT2 with other transporters. | S4–5 |
| 3 | **Fig. S3** The inactivation of *sgvT1.* | S6 |
| 4 | **Fig. S4** The inactivation of *sgvT2.* | S6 |
| 5 | **Fig. S5** The inactivation of *sgvT3.* | S7 |
| 6 | **Fig. S6** HPLC analyses of the fermentation extract of Wild type & Δ*sgvT1.* | S7 |
| 7 | **Fig. S7** HPLC analyses of the fermentation extract of Wild type & Δ*sgvT2.* | S8 |
| 8 | **Fig. S8** HPLC analyses of the fermentation extract of Wild type & Δ*sgvT3.* | S8 |
| 9 | **Fig. S9** HPLC analyses of the fermentation extract of WT::*sgvT1–T2.* | S9 |
| 10 | **Fig. S10** The HPLC standard curve of griseoviridin (GV) /viridogrisein (VG). | S9 |
| 11 | **Fig. S11** HPLC analyses of fermentation extract of complemented mutants. | S10 |
| 12 | **Table. S1** Primer pairs used for PCR-targeting of *sgvT1–T3.* | S11 |
| 13 | **Table. S2** Primers used for PCR confirmation of double-crossover mutants. | S11 |
| 14 | **Table. S3** Primer pairs used for complementation of *sgvT1–T3.* | S11 |
| 15 | **Table. S4** Primer pairs used for RT-PCR. | S11 |
| 16 | **Table. S5** Primer pairs used for qPCR. | S12 |
| 17 | **Table. S6** Quantitative analysis of GV/VG production. | S12 |

10 20 30 40 50 60

---------+---------+---------+---------+---------+---------+

1 -----MTSVRGASKTGRTS--KTSTATTALVLACTAHFLVVFDTSVITVALPSVRADLGF CmcT AL

1 --------MRGS-----------SAARTALALSCTTHFLVLFDVSVMTVALPSIQSDLGF CmcT SC

1 MGQREARAADGSVDTEKETGALVPRQAALLWLACAAQFMVVLDVSVVNVALPSIQTALGF EncT

1 MMQSMSQLLSGK-DGAQEAPSRGGSTWVAVLAACVGQFVVVLDVSVINVALPSIRSGLDI MctT

1 MSQQSSASAPLSPAPPQ----RDPRRWWILAVLCSSLFVIVLDNTILNVAIPSITEQLDA SgvT1

1 MTSTSQEAPRTT----------TSHPRVVLAILCLTQLLLTLDDNVVNIALPTIQRELGF SgvT3

70 80 90 100 110 120

---------+---------+---------+---------+---------+---------+

54 APASLQWVVNSYTLAFAGLLLFGGRLADIHGHRRVFLGGLAVFTLTSLIGGLATSPASLI CmcT AL

42 TPSGLQWVVSAYTLAFAGLLLLGGRLADLYGHRRVFTAGLAVFTVASLLGGLATDPATLI CmcT SC

61 DAAGLQWVVGSYALVFAGFLLLGGRLADLYGRRRAFVWGLALFSVSSLVGGLATGPGLLI EncT

60 GETGLQWVVNAYVIAFAGFLLLGGRASDLFGRKAVFVFGLGVFTAASLLGGLAQAPWMLI MctT

57 STAQIQWTINAYTLVLSGLLITAGGLSDRLGRKRALLLGLLVFGVGSAIGAFAGSPEVLI SgvT1

51 SGPSLVWVVNTYMLAFGGFLVLGGRLGDRYSLVRTFAAGTAVFALASLLSGLAAEPWQLI SgvT3

130 140 150 160 170 180

---------+---------+---------+---------+---------+---------+

114 AARAGQGAGAAVLAPLAVTMLTTSFAEGPRRTRALTISTAVALVGGASGNLLGGVFTEFL CmcT AL

102 AARAAQGLGAAVLAPLSLTMLTTSFPEGPSRTRALTIWTAVALAGGAAGNLLGGLLTEYL CmcT SC

121 AMRAVQGLGAAVLAPATLTILTTTFPEGPGRTKALAIWTAVSSAGGAAGNLIGGVLTDAL EncT

120 VARALQGIGAAVLSPATLAILTTTFPEGPARIKAVAIWTAVGTGGGAAGGLIGGLLTDYL MctT

117 GARALMGVGAAFLMPGTLAVLVRTFDN-EERPKAIGIWAAVSSVGMALGPVLGGFLLDHF SgvT1

111 GARALQGLGAALVAPAVLGLLTLVFTEPAARAKAFAVWGATAVTGALAGLILSGVITEYW SgvT3

190 200 210 220 230 240

---------+---------+---------+---------+---------+---------+

174 SWRSVLLVNVPIGIPVLFLAARVLAGPRKRPWGRVRLDLPGAVLATAGLTLLTLGVSQTH CmcT AL

162 SWRSVLLVNVPVGIPAALFGLRVLAR-RGAPERAVRLDLPGAVLATAALTLLTFGVAQAH CmcT SC

181 SWRWILLINVPIGTVAVLAALRLLPSDRSRAVSG-RLDVPGAVLATLGVTALVYGVNQAG EncT

180 SWRWVLLINVPLGLVVIVATVAWLAESRSDQAHRRRLDLPGAVLVTLGVGSLAYGISQSE MctT

176 WWGVTFLINVPVAVVGLIAMGALLRE--SKDPANARPDLIGALLCTVSVVALVWAVISVP SgvT1

171 SWRWVFFINLPVAALALVLLPRLLRG-IHRPHGDQALNWGGAALLTCGLALLVYALLSFG SgvT3

250 260 270 280 290 300

---------+---------+---------+---------+---------+---------+

234 EHGWGEAAVAVPLAGGLLALLAFVVVEARFAASPLIPPRLFGLPGVGWGNLAMLLAGASQ CmcT AL

221 THAWSDPRTALPLLGGGAALVAFAVVEARFAATPLIPPRLFRLPGIGWGNLAMLLAGASQ CmcT SC

240 TQGWTGATTLTGLAVSAVALTAFAVTESRYTAAPLLPPRLVRLRPIWAGNVTMLLAGGCF EncT

240 GHGWGSPRTLTFLIVGVVALLAFVAVEQR-TREPLMPLGVFRVRSVSAANAITIVSGMGF MctT

234 GQGWLDPETLGAGLLGLAGATVFVRWELR-APDPMLDMTMFKNPKFRGAVFGGILSAFGM SgvT1

230 GDGGRPAVAAACAVLSLALLAAFAVDQRR-SANPLVTKGVFASPVRARALVAAVLLSAAT SgvT3

310 320 330 340 350 360

---------+---------+---------+---------+---------+---------+

294 VPVWFFLTLSMQHVLGYSAAQAGLGFVPHALVMLVVGLRVVPWLMRHVQARVLIAAGAAI CmcT AL

281 VPLWYFLTLTMQDVLGYSAAQTGLGFLPHAAVMLLVSLRLIPLLMRRVQARVLIATGAIV CmcT SC

300 IPMWYFLSLYMQEVLHYGALAAGVGFLPHTLVG-IAAARLAPAVMERTGARGLIVLSAVL EncT

299 YAMWYFLSLYMQNVLKYSAVQTGLALLPHTATI-ILSAQFAPRLMRWIKGRTLLVIAGLL MctT

293 AGSLFLLTQDLQFLRGYAPLEAGAQLTPMAVGVLISSILISPRVVKKLGIGKGVALGVTC SgvT1

289 FQGFLVLTLYLQNGLGFTPLEAGLGYVPFALGSLTGVKAGERATVRFTAGRVLTVACAGT SgvT3

370 380 390 400 410 420

---------+---------+---------+---------+---------+---------+

354 GALGFWWQSLLTPDSAYLGGILGPAVLISIGGGLVGTPLARTVTSGVGPLDAGAASGLMN CmcT AL

341 EAGGFWWQSAITPDSGYVTGILGPAVLLSLGGGLLSTPLTRTVTSGVSHADAGAASGLMN CmcT SC

359 SAVGFVWQSSISTDSGYLDGLLGPAIVMSAGMGLLITPITTTVTSGIREQDAGAASGLMN EncT

358 TAAGFIWQGNMDADGSFLATLLGPGIVFSFGAGLMMTLLAVSATTGVELSESGLVAGLAN MctT

353 SAAG-LLTVALAPDDGYLV-LAAGLLLLGGGAGISGPLVANALMSSIPPERASTGSGVNN SgvT1

349 AAGALLLSWAASREFGYAG-LLPGILLFAIGVGAGLPAAASAAARDVDERTSGVIASLVN SgvT3

430 440 450 460 470 480

---------+---------+---------+---------+---------+---------+

414 TTRQFGGAFGLAVL-LTVTGSGTS---GSPA-ELASHYGDAFVGI--------------- CmcT AL

401 TTRQFGGAFGLAVV-LTLTATGTTGAAGSPA-ALAESYGDAFLSM--------------- CmcT SC

419 TTRQLGGVVGLSAL-VTLATADGG----P-----DLSYRAVFVAT--------------- EncT

418 TSRTMGGALGLSVL-ASVAARRTADVGPGAE-GLASGYGRAFVVS--------------- MctT

411 TLQELGSGLGVGVLGAVLVAAFSQNLPGGLGGAAAESFPAAVAAAGDDSGLLAQVRDSFA SgvT1

408 SSQQLGGAVGLALTGLSLGGSGHG-------------FGAALAVC--------------- SgvT3

490 500 510 520 530 540

---------+---------+---------+---------+---------+---------+

454 ----------AVFMLAIAVLTPVLPALARSTP---PGVIH----------VSPVAR CmcT AL

444 ----------AVILLVLAALTPLLPALRDSRSGARPGAGAGENTDGADGDTRPAVR CmcT SC

454 ----------AAVCAGVAVMALALPAPQEGRD-----------------ESVAMQR EncT

461 ----------GAIILVSMLMIPFLPKPQPQTP---------------------AE MctT

471 DSLTVSQLIGAAAVLLGGWLAGALLLRADRADRARAAAREAEAAVSGSGRADGAADDRPV SgvT1

440 ----------AAAALLACGWAAVVARREPLVS--------------------PSS. SgvT3

| **Protein** | **SgvT1**  **Identity/Similarity** | **SgvT3**  **Identity/Similarity** |
| --- | --- | --- |
| CmcT *A. lactamdurans* | 33/48 | 39/53 |
| CmcT  *S*. *clavuligerus* | 32/50 | 38/52 |
| EncT | 34/49 | 37/52 |
| MctT | 35/52 | 39/55 |
| SgvT1 |  | 31/49 |
| SgvT3 | 31/49 |  |

**Fig. S1** Multiple sequence alignment and homology comparisons for SgvT1 & T3 with other transporters involved in secondary metabolite biosynthesis using Clustal W and NCBI Blast programs, respectively. The conserved transmembrane domains are shaded in gray. The sequences shaded in pink are two different domains with other counterparts. The following proteins and NCBI accession numbers used for the template in alignment including: CmcT (*Amycolatopsis lactamdurans*, Q04733); CmcT (*S*. *clavuligerus*, WP_003952491); EncT (*S*. *maritimus*, AAF81738) and MctT (*S*. *lavendulae*, AAD29366).

10 20 30 40 50 60

---------+---------+---------+---------+---------+---------+

1 MRGRGWRSFSNRGWRRGSQPRAGRGGSLSSRTITGMIEVTDLTKRYG----TTTVVEGLT AvtA

1 ----------------------------MNTQPTRAIETSGLVKVYN----GTRAVDGLD DrrA

1 ----------------MIEVTGLGRVFVKKGRQAKRAKQSGPTAQDS----RVVALDSVN KasK

1 -----------------------------------MIQAKGLAKTFTTKQGPVEAVAGVD MtrA

1 ------------------------------------MTVRGLVKHYG----ETKALDGVD OleC

1 ---MTFACGDTFGEETPMAHAVTAPPGTGGGERAAALAVRGLAKTYP---GGRRALAGVD PdmR1

1 --------------------------------MPSQLTLSHVSKAFG----DHPVLDQVS SgvT2-Fr

1 --------------------------------------GSRVRNALE----RLRRLEEDP SgvT2-Re

70 80 90 100 110 120

---------+---------+---------+---------+---------+---------+

57 FQVRTGR----VTGFLGPNGAGKSTAMRMMLGLDRPTAGEVRIDGAPYQRLRDP------ AvtA

29 LNVPAGL----VYGILGPNGAGKSTTIRMLATLLRPDGGTARVFGHDVTSEPDT------ DrrA

41 LSVPEGE----THGVLGPNGAGKTTLVRILSTMLLPTSGRALVAGHDVTTEADQ------ KasK

26 FTVHTGE----IVGFLGPNGAGKTTTMRMLTTLLPPSSGTAVIAGRDLLHEPAK------ MtrA

21 LDVREGT----VMGVLGPNGAGKTTLVRILSTLITPDSGQATVAGYDVVRQPRQ------ OleC

55 FEVAAGE----VFALLGPNGAGKSTTMRILTTLSRPDAGTATIGGVDVVREPQK------ PdmR1

25 FSIRPGD----TVGVVGENGAGKTTLLRLMAGVEQPDEGTVTLDTEGGVGLLGQTLDLPA SgvT2-Fr

19 VP-RPPDPLRFTAGLAGTGRTFETPLVTLTDVVVGDRLRVGSLSIGQDSRLLVQGPNGAG SgvT2-Re

**Walker A motif**

130 140 150 160 170 180

---------+---------+---------+---------+---------+---------+

107---LR-TVGALLD-ARAVHPGRTALNH-------------LRWLARSNRIP----PRRVR AvtA

79 ---VRRRISVTGQ-YASVDEGLTGTEN-------------LVMMGRLQGYSWARARERAA DrrA

91 ---VRRRIGIVFGGDNGLYTRITARQN-------------LVFWATMYRVDASVVKRRSQ KasK

76 ---VRTHIGYVSQ-AGGAKPSSPVRRD-------------LVLQGRLYGMSRSEAEARAD MtrA

71 ---LRRVIGLTGQ-YASVDEKLPGWEN-------------LYLIGRLLDLSRKEARARAD OleC

105---ARRLIGYVAQ-RSGVDASGTGREN-------------LTLQGRLHGLSGRTLAARVD PdmR1

81 HYTVRDAVDWALADLRETEARLRSLESGMATATRAELDAYGELLHRFEARGGYTADARAA SgvT2-Fr

78 KTTLLDVIAGVLE-PDEGEVVRQGRISYLPQEVSVGKPQQPLLAAFAEGRAGTEEEHR-- SgvT2-Re

**Q loop**

190 200 210 220 230 240

---------+---------+---------+---------+---------+---------+

145 EVIELAGLRSAV--RRRAGTFSLGMSQRLGIAAALLGDPAVLVLDEPVNGLDPEGVLWLR AvtA

122 ELIDGFGLGDAR--DRLLKTYSGGMRRRLDIAASIVVTPDLLFLDEPTTGLDPRSRNQVW DrrA

135 ELLERVGLADRA--DEPVEGFSRGMRQRLHLARGLIGDPAVLFLDEPTMGMDPLATRDFR KasK

119 EVITQLGLEELA--DRIIQTLSGCQARRVDMALGLMHQPELLFLDEPTANLDPESRNDVW MtrA

114 ELLERFSLTEAA--RRPAGTYSGGMRRRLDLAASMIGRPAVLYLDEPTTGLDPRTRNEVW OleC

148 ALLETVGLTEIA--DRGTREYSGGTRRKLDIAMGLVHEPRVLFLDEPTTGLDPEVRAEIW PdmR1

141 AMLDELGIGHIP-FDRPLGALSGGEQSRLALACMLATEPGLLLLDEPSNHLDDRAMAWLE SgvT2-Fr

135 EQLASLGLFDPDTFHVPVGELSVGQQRRLVLARLVVEPADLLLLDEPTNHLALTLLEELE SgvT2-Re

**Walker B motif**

**Signature motif**

**D loop**

250 260 270 280 290 300

---------+---------+---------+---------+---------+---------+

203 HLMRDLAAQG-RTVFVSSHLMSEAALTVDHLVIIGRGRLLADTSMSEFIDRYT--D-VGV AvtA

180 DIVRALVDAG-TTVLLTTQYLDEADQLADRIAVIDHGRVIAEGTTGELKSSLG-SNVLRL DrrA

193 DLVHELKAEG-RTILITTHDMSEAEALCDRVSLVDHGSILVTEPTATIGRMLSGHDRIDV KasK

177 EHIRRLRDERGTTVFLTTHYLDEADSLCDRVLIIDKGRIIAEDSPRSLKAAIS-HDTIEI MtrA

172 DEVKAMVGDG-VTVLLTTQYMEEAEQLASELTVVDRGRVIAKGGIEELKARVG-GRTLRV OleC

206 EHITGLAGAGALTILLTTHYLEEADRLAGRVAILDRGTIVAQGTPAELKGDLR-GDAIHV PdmR1

200 NRLRDFPG----TVVAVSHDRVFLERVTETILEVDGDRHTVNRYGNGYAGYLE------- SgvT2-Fr

195 DALALFPG----AVVIVSHDRRLRSRFRGEPVDL---R----------DGVLR------- SgvT2-Re

**H loop**

310 320 330 340 350 360

---------+---------+---------+---------+---------+---------+

259 RVRTPEPRR-LRDVLVG-AGIT-----VTDCSDGSLKVAAAAER-----IGELVAAHAVT AvtA

238 RLHDAQSRAEAERLLSAELGVT----IHRDSDPTALSARIDDPR-QGMRALAELSRTHLE DrrA

252 TLREDQSP--LLSELAGLPQVER---VDKLSEPGSVRIHTVSAD-ANPVVLRWLLDAGID KasK

236 EVADDTAR--ATELLAAHRDVH-----EVSVNGTVLSISCRDAERLLADLLRTLESARIR MtrA

230 RPVDPLQLRPLAGML-DELGITGLASTTVDTETGALLVPILSDE-QLTAVVGAVTARGIT OleC

265 EVLDPAAAEPARRVLAGLARVR-----EVTVDGVTVRALVEDAAGAVPLVLAALTERGVT PdmR1

249 --EKAAARR----------RWE-------E----AYQNWLDDME------FQRVRSVTTA SgvT2-Fr

231 --RAQAAG----------------------------------------------TSPAAA SgvT2-Re

370 380 390

---------+---------+---------+--------

307 VHEVTRKTVSLEEAFMRLTAAAVEYRAESPGGVRR AvtA

293 VRSFSLGQSSLDEVFLALTGHPADDRSTEEAAEEEKVA DrrA

306 A--LRTGRPTLEEVYVHLVGNRGLTV KasK

289 VESLRVLKPTLDDVFMTLTDRRSQRGADAVHA MtrA

288 LSSITTELPSLDEVFLSLTGHRASAPQDAEPARQEVAV OleC

320 ASSVAVARPSLDDVYLHYAG-RSAKEERA PdmR1

280 HRVGYANRFHSNKLQYFNHGLRAERQV SgvT2-Fr

243 PDA. SgvT2-Re

| **Protein** | **SgvT2-Fr**  **Identity/Similarity** | **SgvT2-Re**  **Identity/Similarity** |
| --- | --- | --- |
| AvtA | 29/44 | 30/42 |
| DrrA | 27/44 | 34/45 |
| KasK | 27/44 | 29/43 |
| MtrA | 32/46 | 33/45 |
| OleC | 30/41 | 31/44 |
| PdmR1 | 30/45 | 28/45 |
| SgvT2-Fr |  | 35/43 |
| SgvT2-Re | 35/43 |  |

**Fig. S2** Multiple sequence alignment and homology comparison of SgvT2 with other transporters involved in secondary metabolite biosynthesis using Clustal W and NCBI Blast programs, respectively. The conserved motifs include the Walker A motif, Q loop, Signature motif, Walker B motif, D loop, H loop and are shaded in gray. The following proteins were used as the template in alignments include: AvtA (*S*. *avermitilis*, WP_010982371); DrrA (*S*. *peucetius*, P32010); KasK (*S*. *kasugaensis*, BAA95019); MtrA (*S*. *argillaceus*, CAK50797); OleC (*S*. *antibioticus*, AAA26793); PdmR1 (*Actinomadura hibisca*, ABM21744).

**Fig. S3** Inactivation of *sgvT1*. (A) Construction of *sgvT1* gene replacement mutant. (B) Gel electrophoresis of PCR products. Δ*sgvT1*: mutant strain; WT: *S.griseoviridis* NRRL 2427; Marker: DNA molecular ladder. Cosmid 4F10 was used to construct pJu5036.

**Fig. S4** Inactivation of *sgvT2*. (A) Construction of *sgvT2* gene replacement mutant. (B) Gel electrophoresis of PCR products. Δ*sgvT2*: mutant strain; WT: *S.griseoviridis*; Marker: DNA molecular ladder. Cosmid 26B3 was used to construct pJu5037.

**Fig. S5** Inactivation of *sgvT3*. (A) Construction of *sgvT3* gene replacement mutant. (B) Gel electrophoresis of PCR products. Δ*sgvT3*: mutant strain; WT: *S.griseoviridis*; Marker: DNA molecular ladder. Cosmid 20F10 was used to construct pJu5038.


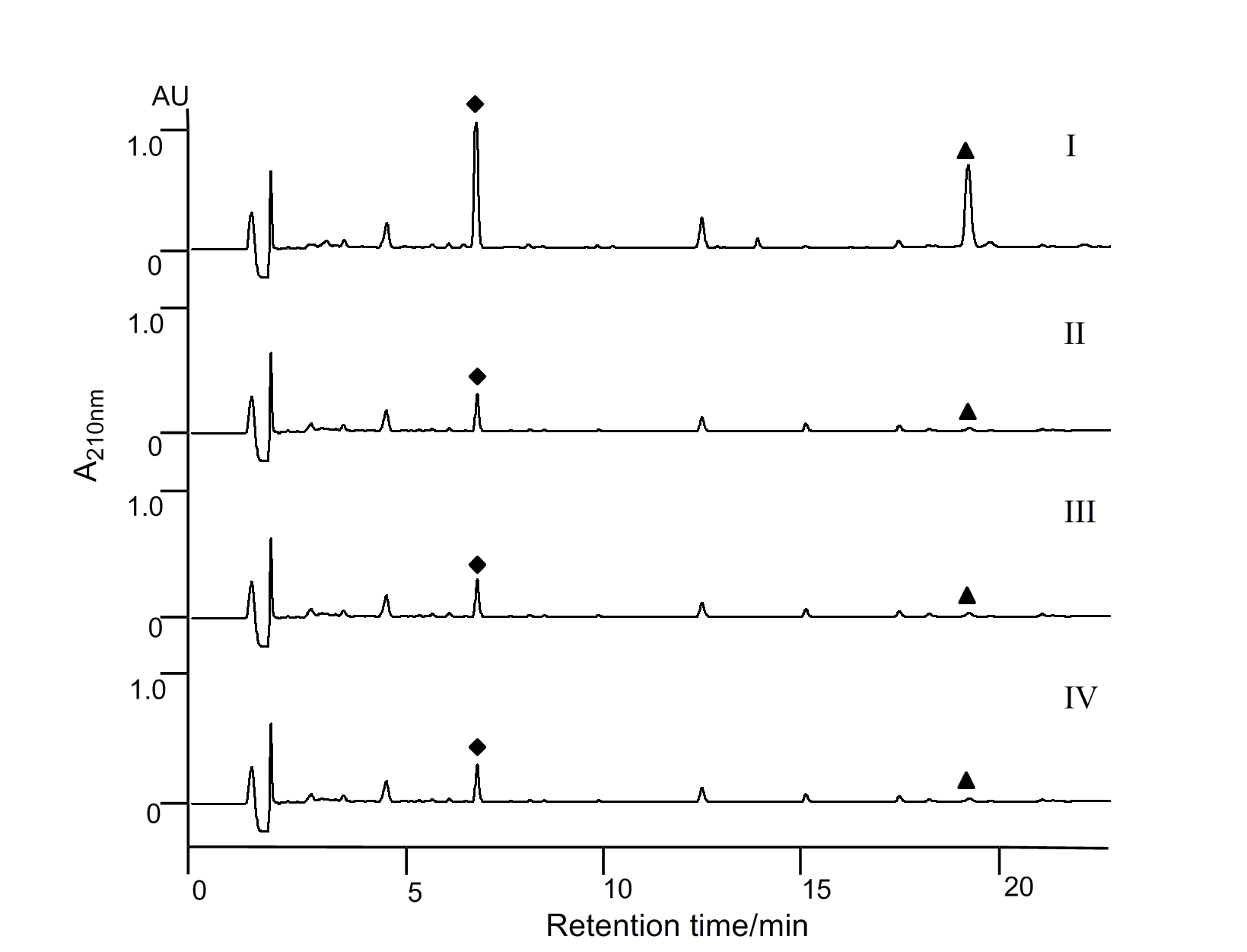


**Fig. S6** HPLC analyses of fermentation extract. *S*. *griseoviridis* NRRL 2427 WT (I); *sgvT1* insertion mutant Δ*sgvT1* (II*–*IV). Symbol code: griseoviridin (GV = ◆); viridogrisein (VG = ▲).

**
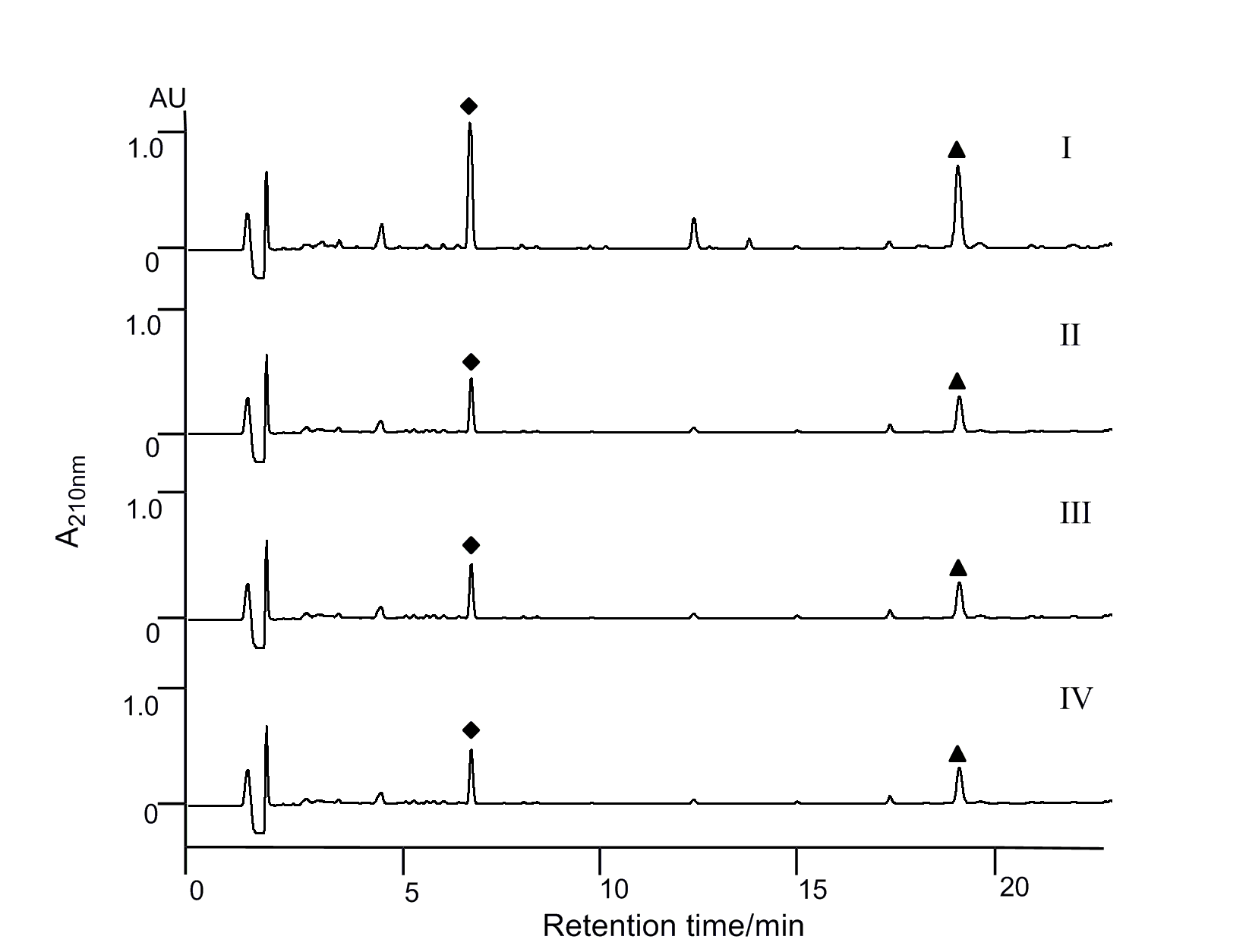
**

**Fig. S7** HPLC analyses of fermentation extract. *S*. *griseoviridis* NRRL 2427 WT (I); *sgvT2* insertion mutant Δ*sgvT2* (II*–*IV). Symbol code: griseoviridin (GV = ◆); viridogrisein (VG = ▲).

**
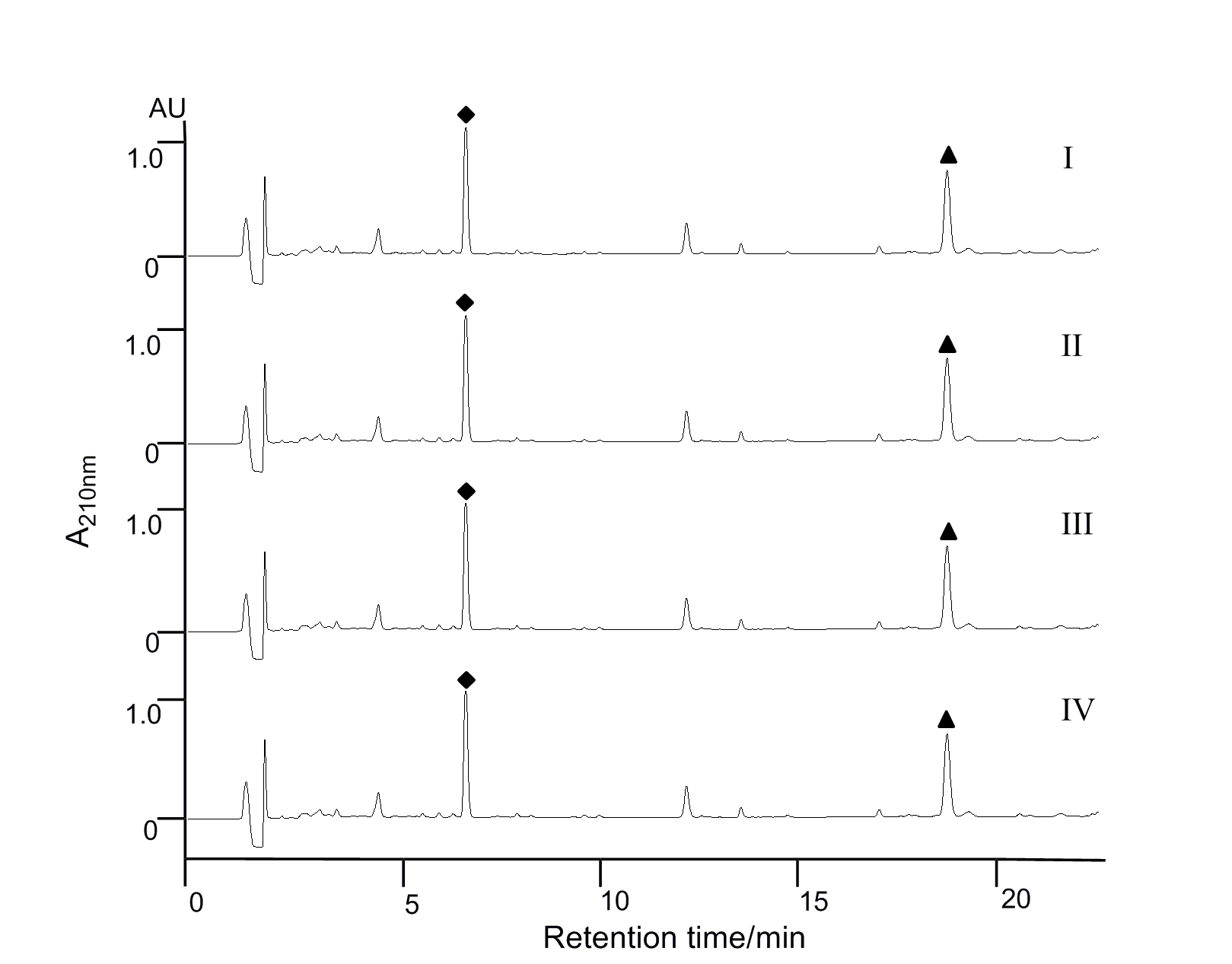
**

**Fig. S8** HPLC analyses of fermentation extract. *S*. *griseoviridis* NRRL 2427 wild type (I); *sgvT3* insertion mutant Δ*sgvT3* (II-IV). Symbol code: griseoviridin (GV = ◆); viridogrisein (VG = ▲).


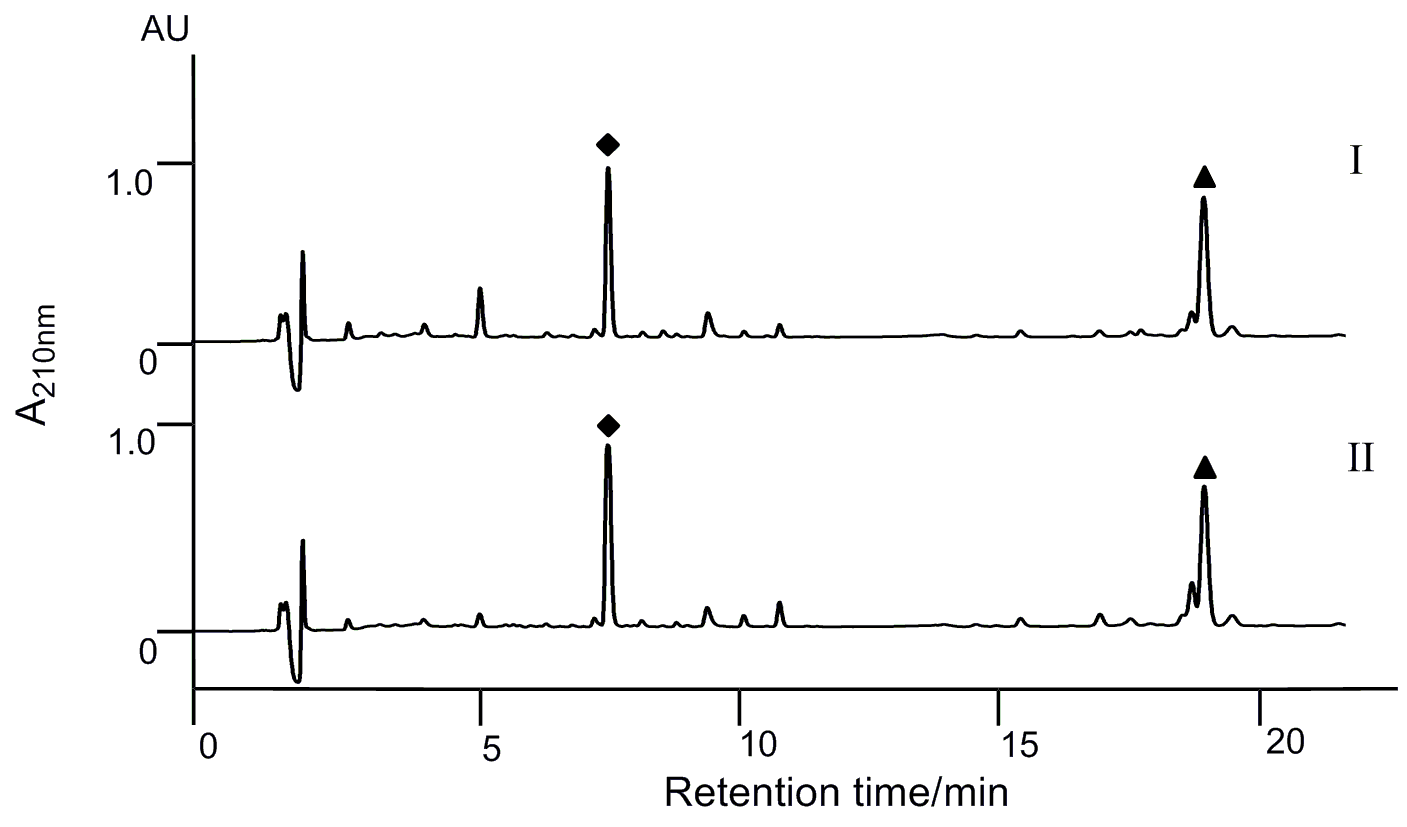


**Fig. S9** HPLC analyses of fermentation extract. 2-fold diluted sample of WT::*sgvT1–T2* (I); 2-fold diluted sample of *WT::sgvT1–T2* which was cultivated with 50 μg/mL apramycin and 50 μg/mL kanamycin. Symbol code: griseoviridin (GV = ◆); viridogrisein (VG = ▲).


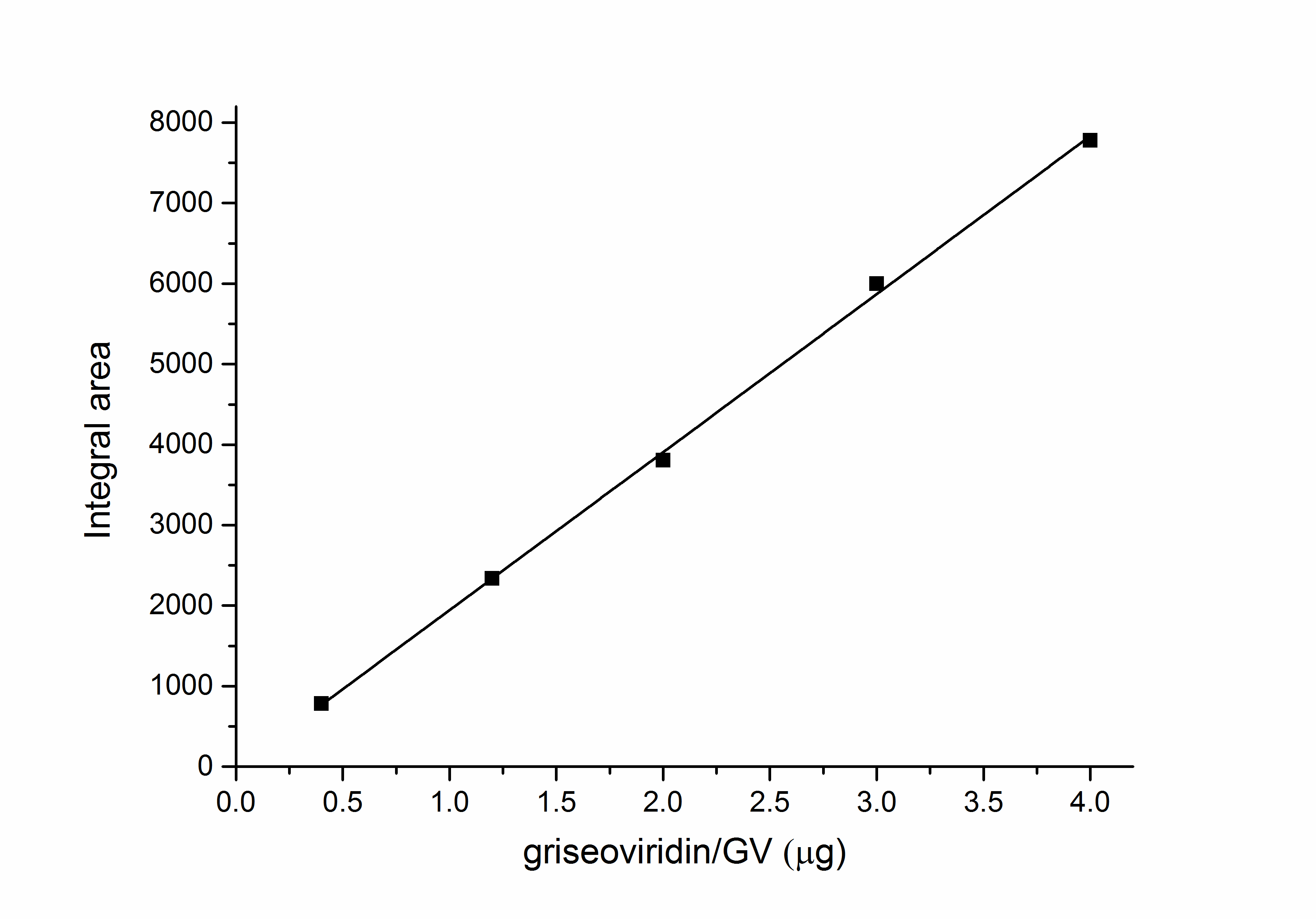

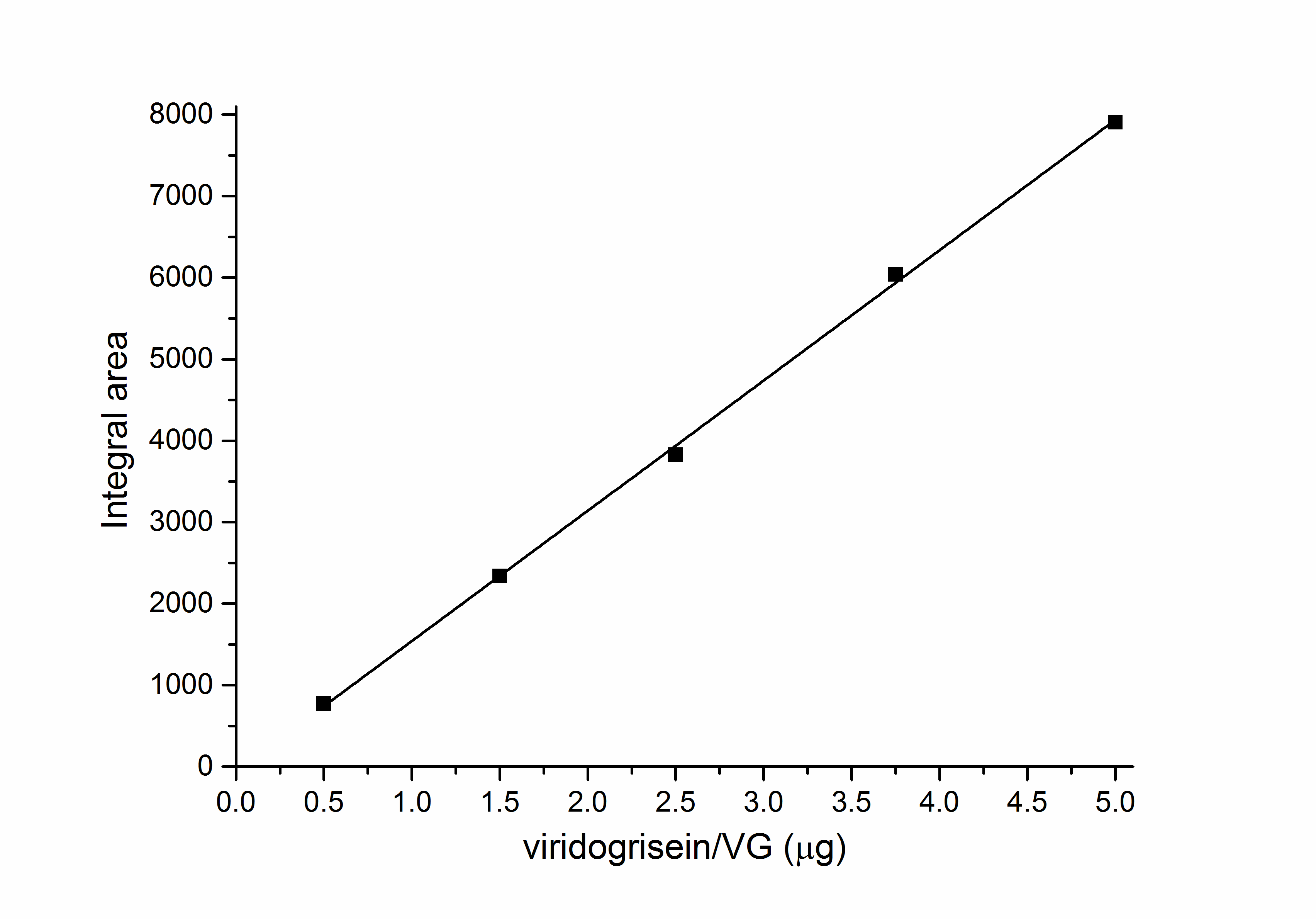


**A**

**B**

**Fig. S10** The quantitative HPLC standard curve for griseoviridin (GV) and viridogrisein (VG). The GV (0.4 mg/mL) curve was analyzed via analysis of a concentration gradient of 0.4 mg, 1.2 mg, 2 mg, 3 mg and 4 mg; VG (0.5 mg/mL) curve was generated via a concentration gradient of 0.5 mg, 1.5 mg, 2.5 mg, 3.75 mg and 5 mg. UV absorption was maintained below 1 A unit to ensure appropriate confidence of the generated standard curve.


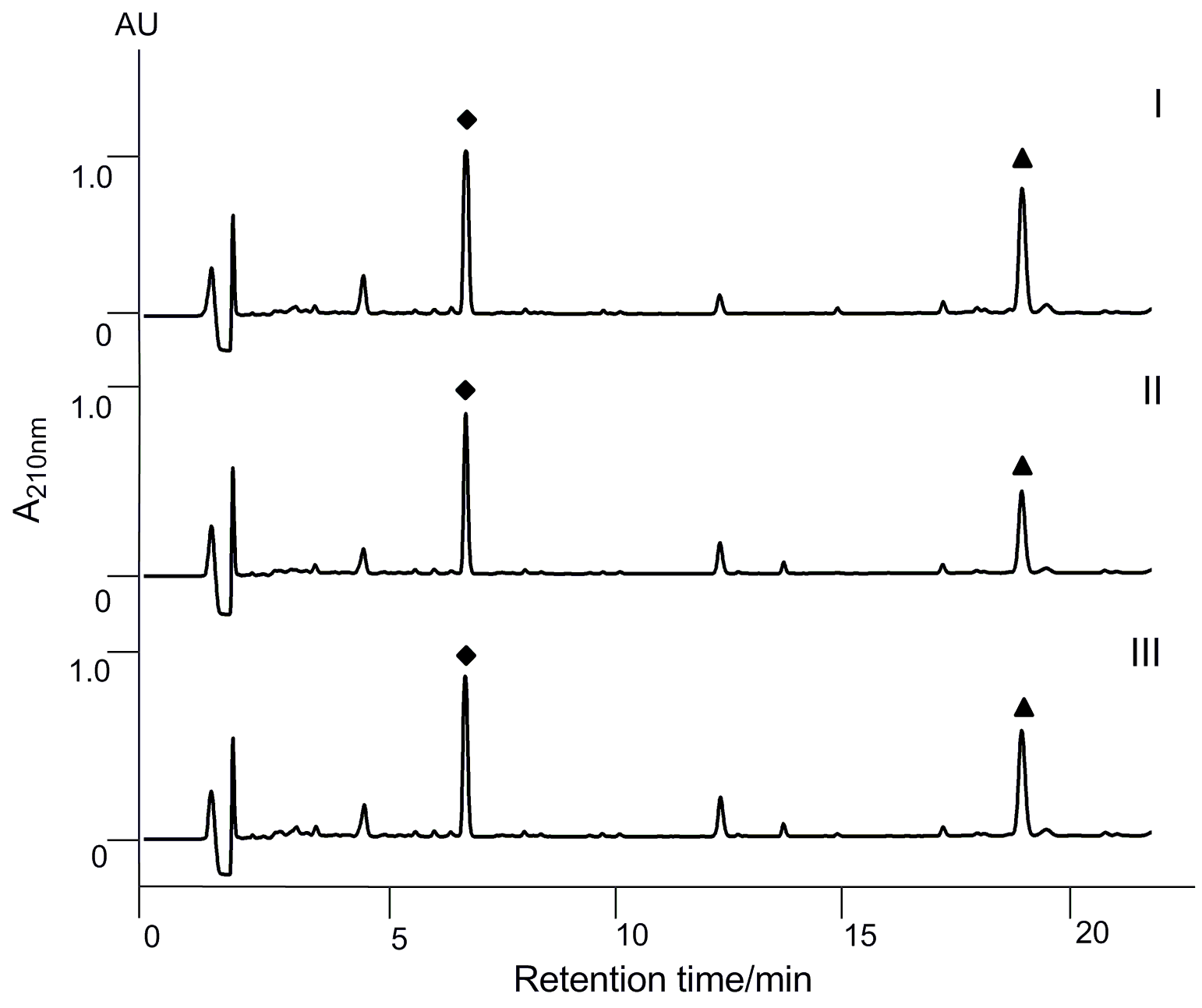


**Fig. S11** HPLC analyses of fermentation extract. *S. griseoviridis* NRRL 2427 WT (I); *sgvT1* insertion mutant Δ*sgvT1* complemented with sgvT1 (II); *sgvT2* insertion mutant Δ*sgvT2* complemented with sgvT2 (III). Symbol code: griseoviridin (GV = ◆); viridogrisein (VG = ▲).

**Table. S1 Primer pairs used for PCR-targeting of *sgvT1–T3**.**

| Gene | Primer pairs used for inactivation (5’→3’) |
| --- | --- |
| *sgvT1* | sgvT1delF:CTGTCGCCGGCACCTCCCCAGCGCGACCCCCGCCGCTGGattccggggatccgtcgacc  sgvT1delR: GGCCGCTTCGGCCTCCCGCGCCGCGGCCCGCGCGCGGTCtgtaggctggagctgcttc |
| *sgvT2* | sgvT2delF: CCGTGCTCGACCAGGTCTCCTTCAGCATCCGTCCCGGCattccggggatccgtcgacc  sgvT2delR: CAGCAcCCCGTCCCGCAGGTCCACCGGCTCGCCGCGGAAtgtaggctggagctgcttc |
| *sgvT3* | sgvT3delF: GTCCTGGCCATCCTCTGCCTCACCCAGCTCCTGCTGACCattccggggatccgtcgacc  sgvT3delR: GGCCAGCAGCGCGGCGGcGGCGCaCACCGCCAGGGCCGCtgtaggctggagctgcttc |

*Capital letters were derived from targeted genes

**Table. S2 Primers used for PCR confirmation of the double-crossover mutants.**

| Gene | Primer pairs designed to verify the mutant strains (5'→3') | Fragment replaced  (bp) | Length of desired PCR fragments | |
| --- | --- | --- | --- | --- |
|  |  |  | Wild strain (bp) | Mutant strain (bp) |
| *sgvT1* | sgvT1F: TCCGGAAGGCCACTCCCAAC  sgvT1R: GTGCGTTCGACACCCCGACC | 1434 | 1708 | 1643 |
| *sgvT2* | sgvT2F: GATGTCCCAGTCCCCCACAG  sgvT2R: CACCGGTACCCCGGTGGGTC | 1476 | 1849 | 1742 |
| *sgvT3* | sgvT3F: CTTGCGGACCGCGCGTACGC  sgvT3R: TCTGGACGGCGACGGAGAG | 1206 | 1547 | 1710 |

**Table. S3 Primer pairs used for complementation of *sgvT1*-*T3.***

| Gene | Sequence (5’→3’) |
| --- | --- |
| *sgvT1* | sgvT1CF: **CATATG**TCCCAGCAGTCGTCAGCGTCAGCC  sgvT1CR: **TCTAGA**TTAGACCGGGCGGTCGTCCGCGGC |
|  | sgvT1C2F: **CATATG**TCCCAGCAGTCGTCAGCGTCAGCC  sgvT1C2R: **ACTAGT**CTAGACCGGGCGGTCGTCCGCGGC |
| *sgvT2* | sgvT2CF: **CATATG**CCTTCTCAACTCACGCTTTCCCAC  sgvT2CR: **TCTAGA**TTAGGCGTcGGGTGCGGCGGCCGG |

*Primer pairs of sgvT1C and sgvT1C2 were used to amplification for ligation with pSET152AKE and pPWW50Apr two vectors respectively.

*The bold letters represent the designed enzyme digestion site.

**Table. S4 Primer pairs used for RT-PCR.**

| Gene | Sequence (5’→3’) | Amplified length(bp) |
| --- | --- | --- |
| *sgvT1* | sgvT1rtF: TTCCTGGAGGGTGTTGTTGACG  sgvT1rtR: ATGGCCGGTTCGCTGTTCC | 369 |
| *sgvT2* | sgvT2rtF: CGGGGTACCTGGAGGAGAAGG  sgvT2rtR: CACGTCCGTCAGCGTCACCAG | 335 |
| *sgvT3* | sgvT3rtF: GCCCACCATTCAGCGGGAAC  sgvT3rtR: CGACCGGCAGGTTGATGAAG | 425 |
| *orf(+2)* | orf+2rtF: CACCGCCGTACCAGGGACAGG  orf+2rtR: CATGCTCGCCGAGGGCATCAC | 396 |

**Table. S5 Primer pairs used for qPCR.**

| Gene | Sequence (5’→3’) | Amplified length(bp) |
| --- | --- | --- |
| *sgvA* | sgvAqF: AGCACCAGTTCCTGATGT  sgvAqR: CGTATCACCATGTCGTAGT | 157 |
| *sgvR1* | sgvR1qF: GGTCTCGAAGTGGAAGTG  sgvR1qR: TACCAGATGGCTGCTGAT | 130 |
| *sgvR2* | sgvR2qF: CCGTGTTCAGCATGTATCC  sgvR2qR: GCAGATCCGCGAACTGAT | 128 |
| *sgvR3* | sgvR3qF: AGATGTGCCGACAAGGAC  sgvR3qR: GCGATGACGTTCTGATGC | 200 |
| *sgvD1* | sgvD1qF: CCACGACGACTACCTGTA  sgvD1qR: GATCAGCTCGAAGCACTC | 196 |
| *sgvE1* | sgvE1qF: TTCTGGGACCTCCTCGTA  sgvE1qR: CGCTATGCCGAAGAACTC | 156 |
| *sgvT1* | sgvT1qF: TGTGTAGGCGTTGATCGT  sgvT1qR: GTGATCGTGCTGGACAAC | 105 |
| *sgvT2* | sgvT2qF: GAACTGGCTGGACGACAT  sgvT2qR: GTACTGGAGCTTGTTGGAG | 100 |
| *sgvT3* | sgvT3qF: CGACAACGTCGTCAACAT  sgvT3qR: GAAGGCCAGCATGTAGGT | 100 |

| **Sample** | **Time (h)** | **GV (μg/mL)** | **VG (μg/mL)** |
| --- | --- | --- | --- |
| WT | 108 | 33.04 ± 0.70 | 31.56 ± 0.51 |
| WT supernatant | 108 | 24.54 ± 0.25 | 24.56 ± 0.37 |
| WT intracellular | 108 | 8.12 ± 0.62 | 7.12 ± 0.52 |
| WT::*sgvT1*-*T2* | 120 | 106.85 ± 1.81 | 106.61 ± 1.12 |
| Δ*sgvT1* | 60 | 5.28 ± 0.19 | 4.65 ± 0.13 |
| Δ*sgvT1* intracellular | 60 | 0.77 ± 0.01 | 0.67 ± 0.07 |
| Δ*sgvT2* | 108 | 10.05 ± 0.09 | 11.21 ± 0.35 |
| Δ*sgvT2* intracellular | 108 | 1.22 ± 0.14 | 1.06 ± 0.21 |

**Table. S6** Quantitative analysis of GV/VG production.
